# Dietary potassium liberalization with fruit and vegetables versus potassium restriction in people with chronic kidney disease (DK-Lib CKD): a clinical trial protocol

**DOI:** 10.1186/s12882-023-03354-4

**Published:** 2023-10-13

**Authors:** Yasmin Iman, Robert Balshaw, Mackenzie Alexiuk, Jay Hingwala, Leah Cahill, Rebecca Mollard, Navdeep Tangri, Dylan Mackay

**Affiliations:** 1 Chronic Disease Innovation Centre, Winnipeg, MB Canada; 2 Department of Community Health Sciences, University of Manitoba, Max Rady College of Medicine, Winnipeg, MB Canada; 3 Department of Internal Medicine, University of Manitoba, Max Rady College of Medicine, Winnipeg, MB Canada; 4 Department of Medicine, Dalhousie University, Halifax, NS Canada; 5 Faculty of Agriculture and Food Sciences, Department of Food and Human Nutritional Sciences, University of Manitoba, Manitoba, Canada; 6 George and Fay Yee Centre for Healthcare Innovation, Winnipeg, MB Canada

**Keywords:** Chronic kidney disease, Potassium, Hyperkalemia, Nutrition, Quality of life

## Abstract

**Background:**

Potassium regulation in the body is primarily done in the kidney. In addition to this, hyperkalemia, occurs in approximately 10% of individuals with chronic kidney disease (CKD) and is associated with elevated all-cause mortality. Individuals with CKD are often told to restrict dietary potassium (K), however, this recommendation is based on low quality evidence. Reduced quality of life, limited dietary choices and nutritional deficiencies are all potential negative outcomes that may occur when restricting dietary K in CKD patients. There is a need for randomized controlled trials investigating the impact of dietary K modification on serum K concentrations in people with CKD.

**Methods:**

A randomized 2-period crossover design comparing a liberalized K fruit and vegetable diet where participants will be required to consume ~ 3500 mg of dietary K daily, to a standard K restricted diet where participants will be required to consume < 2000 mg of dietary K daily. All participants will begin on a liberalized K run-in period for 2 weeks where they will receive fruit and vegetables home deliveries and for safety will have clinical chemistry, including serum potassium measurements taken after 1 week. Participants will then be randomized into either liberalized K or standard K diet for six weeks and then crossover to the other intervention for another 6 weeks after a 2-week washout period.

**Discussion:**

30 male and female CKD outpatients, ≥ 18 years of age, who have an estimated glomerular filtration rate (eGFR) between 15 and 45 ml/min/1.73m^2^ and serum K between 4.5 and 5.5 mEq/L. This design would have greater than 80% power to detect a difference of 0.35 mEq/L serum K between groups. Anthropometric measurements, clinical chemistry, dietary recalls, physical function assessments, as well as a quality of life assessments will also be measured in this trial. These findings will provide high quality evidence for, or against, recommendations for dietary K restriction in individuals living with CKD. The removal of K restriction could provide individuals living with CKD more dietary choice leading to improved dietary status and quality of life.

**Trial Registration:**

This trial has received approval from the University of Manitoba Research Ethics board (HS25191 (B2021:104)).

**Supplementary Information:**

The online version contains supplementary material available at 10.1186/s12882-023-03354-4.

## Introduction

### Background and rationale

Chronic kidney disease (CKD) is a major public health concern with rising incidence and prevalence worldwide [1]. More than one in ten Canadians suffer from CKD, and it is currently a leading contributor to global morbidity, mortality, and increased healthcare costs [2, 3]. To help slow CKD progression, optimal management includes interventions such as blood pressure control, RAASi, and SGLT2i therapy [4, 5]. Equally as important are management of its complications, which include anemia, metabolic acidosis, mineral bone disorder and hyperkalemia [6].

The kidney is the primary site of potassium (K) regulation in the body. Hyperkalemia, elevated serum K (sK) occurs in approximately 10% of individuals living with CKD and is associated with elevated all-cause mortality. Hyperkalemia may result from many factors that influence both internal and external K regulation. Comorbid conditions commonly found in patients with CKD such as diabetes mellitus and metabolic acidosis, as well as the use of RAASi and K sparing diuretics, are associated with hyperkalemia [7]. Potassium binders exist, but they are poorly tolerated, can cause rare but fatal gastrointestinal side effects [8], have insufficient long-term data and there is poor access for most Canadians due to cost [9]. Therefore, to minimize the risk of hyperkalemia, individuals living with CKD are often recommended to restrict dietary K (dK). A systematic review and meta-analyses done in 2019 showed that the recommendation to restrict dietary K is based on very-low quality evidence, mostly epidemiological studies and a single randomized controlled trial (RCT) involving individuals living with end stage renal disease (ESRD) who received only manufactured liquid diets with low K concentrations [10]. While the review authors concluded that it is prudent to continue to restrict dK based on low-quality evidence, definitive trials are needed to determine if dK restrictions lowers sK [10].

Diets rich in fruit and vegetables are also typically rich in K as well as other minerals, vitamins and fiber, and are a source of dietary bicarbonate [11]. High K diets, where the K is from fruit and vegetables, are associated with health benefits, including reduced risk of cardiovascular disease [12]. Regrettably, due to the K content fruit and vegetable intake is often reduced when dK is restricted. The benefit of restricting high K fruit and vegetables in terms of sK lowering may not be guaranteed because high K fruit and vegetables often contain carbohydrate, which promotes insulin secretion and K uptake into cells, therefore the impact of different sources of dK influence sK concentrations is not always predictable. Additionally, dK restrictions can negatively impact quality of life, dietary choices and may put participants at risk of nutritional deficiencies such as decreased calcium, therefore it is essential that such a restriction should be based on good quality evidence [13]. There is a clear need for high quality RCTs investigating the impact of dK modification on sK concentrations in individuals living with CKD.

### Objectives

The objectives of this trial are to evaluate the impact and safety of dK liberalization with fruit and vegetables on sK concentrations and to monitor the effects it may have on their physical function and overall quality of life in patients with CKD.

For this trial, our primary hypothesis is that liberalization of dietary K intake, via the provision of higher K fruit and vegetables will be non-inferior to the standard recommended CKD diet in terms of change in sK concentrations. We also hypothesize that liberalization of dietary K will improve self-reported quality of life and lower blood pressure.

### Trial design

This study is an open-label, randomized 2-period crossover definitive superiority trial. The study will be sixteen weeks in duration. Thirty male and female individuals living with CKD will be recruited, with a minimum 40% of each sex. Participants will be randomized to receive both restricted (standard care) and liberalized (experimental) K content fruit and vegetables for six weeks.

## Methods: participants, interventions and outcomes

### Study setting

This clinical trial will take place in Winnipeg, Manitoba, Canada. Recruitment of patients will happen from renal clinics at both Health Sciences Centre and Seven Oaks General Hospital. This trial has the potential to lead to beneficial changes in dietary recommendations for K intake among individuals with CKD in Canada.

### Eligibility criteria

The inclusion criteria include individuals who are over the age of 18, have an eGFR between 15 and 45 ml/min/1.73m^2^, have serum potassium levels between 4.9 and 5.5 mEq/L and a hemoglobin A1C ≤ 11%. Furthermore, participants included in this study must be registered at a multidisciplinary nephrology clinic in Winnipeg and must be able to communicate in English in order to provide written informed consent.

Individuals will be excluded from the study if:

- Serum potassium > 5.5 or < 4.9 mEq/L.
- They have anuria, are on dialysis or have had acute kidney injury failure within six months prior to screening.
- Chronic obstructive pulmonary disease that requires one to be on oxygen.
- Have had a myocardial infarction or stroke in the past 6 months.
- Are currently on potassium binding therapy.
- Have class 3–4 heart failure symptoms or have had their liver, heart or kidneys transplanted.
- Unable to consume study treatments or control, such as swallowing or GI issues.
- People who are pregnant or lactating.
- Any individuals in the opinion of the investigator with any medical condition, uncontrolled systemic disease or concurrent illness that would decrease the study compliance or jeopardize the safety of the participant.

Once participants have been consented they will undergo a baseline study visit to confirm their eligibility to be enrolled into the study. During the baseline visit, anthropometric measures and blood pressure in triplicate will be collected. Participants will also be asked to complete the Kidney Disease Quality of Life Short Form (KDQOL-SF) questionnaire and the Automated Self-Administered 24-hour Canada (ASA24®) dietary assessment tool prior to being randomized to an intervention (Appendix B and Appendix C). Additionally, participants will then undergo a five repetition stand time (STS5) test that will be utilized to assess their physical function in regard to strength, mobility, balance and fall risk. Information on concomitant medications or supplements information will be obtained from the participant’s clinical records. Clinical chemistry, such as HbA1c, albumin, bicarbonate, calcium, sodium, potassium, chloride, creatinine, glucose, phosphate, as well as urea, urine albumin creatinine ratio and urine electrolytes. These will be collected and analyzed by Shared Health Manitoba which includes both blood and urine sampling. Once eligibility is verified and the participant is enrolled, they will be placed on the liberalized dK + via fruit and vegetables diet run-in for 2 weeks (intervention described below), prior to randomization.

A safety assessment will be conducted one week after baseline to ensure participants meet the eligibility criteria and that it is safe for them to continue on to the randomization stage.

## Interventions

### Intervention descriptions

Once the baseline, run-inand safety assessments have been completed, participants will be randomized into one of the two interventions:

*Liberalized dK + via fruit and vegetables (Experimental group)*.

This group will receive weekly supplementation of higher K fruit and vegetables via home delivery during the liberalized dK run in and treatment period. The weekly deliveries will contain combinations of fresh, frozen and dried fruit and vegetables, as well as juices and low-sodium soups which have been selected for their K content and shelf-life. Contents may vary throughout the intervention period based on price and availability. Participants will receive weekly deliveries of K rich fruit and vegetables that contain more than 250 mg of K per 100 g serving, such as potatoes, legumes, beets, carrots, oranges and pears. prior to the run-in week, participants will receive a 30-60-minute counseling session from the study registered dietitian (RD), where they will be instructed to incorporate the fruit and vegetables into their diet targeting a daily dK intake of 2000 mg from the delivered fruit and vegetables over 3 to 4 meals, and a daily dK intake of approximately 3500 mg. During this counseling session from the study RD standard renal diet with instructions to avoid none fruit and vegetable sources of dK will be reinforced, but not the preparation of vegetables to reduce dK content.

#### Standard dK restriction (standard care group)

Participants allocated to this group will receive weekly supplementation via home delivery of lower K fruit and vegetables, that deliver less than 200 mg of K per 100 g serving, such as cauliflower, lettuce, cabbage and blueberries. Prior to starting this intervention participants will receive a 30-60-minute counseling session from the study registered dietitian (RD), where they will be instructed to incorporate the fruit and vegetables into their diet targeting a daily dK intake of 500 mg from the delivered fruit and vegetables over 3 to 4 meals and a daily dK intake less than 2000 mg. During this counseling session from the study RD standard renal diet with instructions will be reinforced, including the preparation of vegetables to reduce dK content.

### Criteria for discontinuing or modifying allocated interventions

Each participant has the right to withdraw from the trial at any time. Participants may discontinue trial participation at any time and are requested to contact a research team member to inform them of their decision. In addition, the Investigator may discontinue a participant from the trial at any time if the investigator considers it necessary for any reason including:

- Pregnancy.
- Ineligibility.
- Significant protocol deviation.
- Significant non-compliance with the protocol.
- Disease progression which results in inability to continue with the protocol.
- Withdrawal of consent.
- Loss to follow-up.
- Significant elevated risk due to hyperkalemia.

Withdrawal will not result in exclusion of the data for that participant from analysis. As the primary analysis will be based on an intention-to-treat there will also be a completer only analysis performed. If the participant is withdrawn within the first two weeks of the trial they will be replaced. If the replacement participant is withdrawn there will be no subsequent replacement. The reason for withdrawal will be recorded in the case report form (CRF), if provided.

### Strategies to improve adherence to interventions

Participants enrolled in both intervention groups will be provided with weekly home delivery of fruit and vegetables to be used in the intervention. There will also be weekly check-ins via email or phone calls to participants regarding the weekly delivery of the fruit and vegetables where coordinators will make sure the delivery contents are acceptable to the participant, with modifications to the delivery contents made according to participant feedback in order to enhance adherence.

### Relevant concomitant care permitted or prohibited during the trial

All participants enrolled in the study will continue to receive multidisciplinary CKD care during and after the trial. Patients that are already on potassium binding therapy will be excluded from this trial. If it is determined that a participant needs to start potassium binding therapy they will be withdrawn from the trial and the reason for withdrawal will be recorded.

### Outcomes

The primary outcome is evaluating sK concentration during the five timepoints at week 0, 1–2, 8, 10 and 16. The secondary outcomes include assessing health related quality of life through KDQOL-SF and STS5 which will be assessed throughout the trial, analyzing clinical chemistry systolic and diastolic blood pressure, and assessing dietary data using the information received from ASA24. Clinical chemistry will look at sodium, potassium, urea, phosphate, and the albumin/creatinine ratio in the urine alongside the collection of blood samples for serum potassium, hemoglobin A1C, albumin, bicarbonate, calcium, chloride, creatinine, eGFR, glucose, phosphorus, sodium and blood urea nitrogen measurement. All samples will be collected and processed by the Diagnostic Services of Manitoba. Individuals will be scheduled for an in-person visit which will include a clinical exam and blood sample to monitor serium potassium and bicarbonate during weeks 1 or 2. Secondary outcome measures will be measured on week 0, 8, 10 and 16. Additionally, anthropometric measurements such as height, weight and BMI will be collected at baseline alongside blood pressure in triplicate.

### Participant timeline

See Appendix A.

For the first two weeks, all participants will start on a liberalized potassium run-in period. Following the run-in period, the participants will be randomized to either the experimental group or the standard care group for six weeks. After the six weeks, a crossover will occur after a two-week washout period.

### Sample size

It has been estimated that a sample size of thirty individuals with each sex will provide 80% power to detect a minimal clinically important difference of 0.35mmEq/L, the reduction shown with a starting dose of Patiromer (4.2 g twice daily) in a double-blind placebo-controlled trial of patients with CKD and hyperkalemia [14]. This assumes a within-subject standard deviation of 0.46, based on a 95% confidence interval of (0.38, 0.72) mEq/L seen in a similar 29-participant cross-over study unpublished, summary results of NCT00949585 found in appendix of Marklund et al [15].

### Recruitment

The recruitment for this study will be done by the research coordinator who will work directly with a healthcare professional (such as a nurse or physician) within the participants circle of care. The healthcare professional will help identify potentially eligible participants. If the participant consents to be contacted, the research coordinator will meet the participant through a virtual meeting or an in-person visit to collect informed consent to participate in the study. A total of thirty participants will be recruited to participate in this study, from Seven Oaks Hospital and Health Sciences Centre renal clinics in Winnipeg, Manitoba. The recruitment period is targeted to last thirty weeks, with enrolment predicted to go at a rate of one participant per week.

## Assignment of interventions

### Sequence generation

In order to ensure allocation concealment, balance and to minimize bias, randomization will be performed by a third-party biostatistician at the George and Fay Yee Centre for Health Innovation. Randomization will be performed using code written in the R statistical programming language (Versions 3.5.3). Treatments will be assigned with a 1:1 ratio of liberalized dK then standard dK or standard dK then liberalized dK, stratified by sex, with random block sizes of 2.

### Concealment mechanism

Allocation to intervention group will be perfomed by a trial coordinator using a secure, web-based REDCap tool hosted at the University of Manitoba using the tables created in R. To minimize bias, intervention assignments will not be released until run-in period completed.

### Implementation

The study biostatistician will generate the allocation sequence and assign participants to the interventions. The study coordinator will enroll the participants.

### Blinding

Assessors who will be completing all assessments will be blinded to the intervention assignment. We will ask participants not to communicate their intervention to the assessor. Blinding of the participants and study staff is not possible given the nature of the intervention, but the study statistician and the entire data management team will be blinded to allocation during analysis.

## Data collection, management and analysis

### Data collection methods

Data will be entered into REDCap database on a secure REDCap server housed by the University of Manitoba. Routine data management audits and data quality checks will be conducted by individuals in the Data Management group at the George and Fay Yee Centre for Health Innovation.

All research records will be kept for 10 years. After 10 years, paper files will be disposed of using the confidential document destruction method at the Chronic Disease Innovation Centre. The electronic data that will be obtained from the trial will be retained and de-identified for a total of 10 years once the study has been completed. The electronic trial data that was obtained from the trial may be utilized in academic journals for publication, however the data will be in shared in a de-identified format. Additionally, the electronic data may potentially be stored by open-access repositories or academic journals under an open access policy which may allow different research teams to access the data for research purposes and further data analysis [16].

### Data Management

A unique numerical coding system that will not contain any direct identifiers, such as name or initials to de-identify the data collected in this study. Data will be entered into REDCap database on a secure REDCap server housed at University of Manitoba. The Data Coordinating Centre that is located within the Data Science group at the Centre for Healthcare Innovation will be taking on the REDCap database data management. They will also be conducting regular data quality checks to ensure data is well kept. A unique code will be utilized for input into the study database for all research data collected throughout the duration of the trial. To ensure the following of site protocols, all research records will be kept for 10 years, following the 10 years, the confidential document destruction method will be used to dispose of any paper files [16]. Additionally, electronic data will be retained and de-identified for 10 years once the trial has been completed.

### Statistical methods

The primary analysis will be based on sK concentration, comparing the endpoints of the intervention periods. The effects of treatment on primary and secondary outcomes using a linear mixed-effects model for the repeated measures will be examined (essentially a paired comparison of the endpoints of the standard care dK diet versus the experimental dK diet). Sex will be included in this model as a fixed factor. Model assumptions for outcome data will be verified by visual inspection of residual plots and through sensitivity analyses regarding missing value strategies. Outcomes which appear to violate the modelling assumptions may be transformed prior to analysis or analysed using another, more appropriate model (e.g., generalized linear mixed models) in supportive analyses. Demographic data will be reported as the mean ± standard deviation. Analyses of the secondary outcomes will be reported as unadjusted and adjusted (expected marginal) means with 95% confidence intervals.

The primary analysis will be conducted using the All Participants (intent to treat) analysis set. The primary analysis will be repeated in the Completers analysis set. Demographics and all other baseline measurements will be analyzed in the All Participants set as well as in the Completers set.

The number and proportion of missing values will be documented in the clinical study report. Missing values will not be imputed unless otherwise noted. Analyses will exclude data from participants who have missing values for any variable required for the analysis. When data are observed to be unusual in a way that cannot be explained or ruled to be in error, analyses may be repeated after excluding the record involved. These additional analyses will be presented as sensitivity analyses. Additionally, all protocol deviations documented in the clinical trial database will be tabulated (if appropriate) and listed in the clinical study report. In general, analyses resulting in p-values less than 0.05 will be described as statistically significant, without adjustment for multiple inference. All results arising from secondary, exploratory and supportive analyses will be identified as such. Overall, the analysis done in this trial is the similar to the ReDACKD clinical trial [16].

## Monitoring

### Data safety monitoring committee

A data safety monitoring board (DSMB) will be formed that include two nephrologists from outside the study team, as well as an independent statistician. The DSMB will conduct a safety review of the study when half the participants have finished their first run-in period and then every 3 months until trial completion. The DSMB will be primary focused on participant safety, but will also review study progress and conduct. As this is a trial there will be no prespecified stopping criteria. As this is a short-term crossover trial there will be no prespecified stopping criteria.

### Interim analyses

The trial will be continued until all recruited participants have reached the end of their follow-up and data have been collected, processed and cleaned. There are no plans for early termination unless a pattern of hyperkalaemia is seen in the run-in period and the DSMB and or qualified investigator recommends early termination due to risk.

### Harms

All adverse events (AE) occurring during the trial that are observed by the Investigators or reported by the participant will be recorded on the CRF, whether or not attributed to trial intervention. The following information will be recorded: description, date of onset and end date, severity, assessment of relatedness to trial intervention. Follow-up information should be provided as necessary. In case any adverse event is reported, patients will be offered to be seen in the next available clinic visit or within one week, whichever is earlier, and will continue to be followed in the clinic until the AE is resolved [16]. The severity of events will be assessed on the following scale: 1 = mild, 2 = moderate, 3 = severe. AEs considered related to the trial intervention as judged by the Qualified Investigator will be followed either until resolution or the event is considered stable. In case AEs result in withdrawn from the trial, the patients that are withdrawn due to adverse treatment reaction will also be followed by the CKD clinic until the AEs has resolved [16].

### Monitoring of trial conduct

Regular monitoring will be performed according to good clinical practices (GCP) by the principal investigators or a delegate. A subset of all data will be evaluated for compliance with the protocol and accuracy in relation to source documents. Following written standard operating procedures, the monitoring will verify that the clinical trial is conducted and data are generated, documented and reported in compliance with the protocol, GCP and the applicable regulatory requirements [16].

## Ethics and dissemination

### Research ethics approval

DK-Lib CKD has received approval from the University of Manitoba Health Research Ethics Board (HS25191 (B2021:104)). All amendments to this studies protocol are reviewed and approved by the University of Manitoba Biomedical Research Ethics Board and changes to the protocol are updated on clinicaltrials.gov (NCT05090865).

### Protocol amendments

The University of Manitoba Research Ethics Board will be responsible for approving and signing off the trial protocol and any protocol amendments. Before any participants are enrolled into the study, the PI at each site is required to obtain local approvals and agreements with Shared Health Manitoba and the University of Manitoba.

### Who will take informed consent

The participant must personally sign and date the latest approved version of the Informed Consent form through the REDCap online platform before any trial specific procedures are performed. Informed consent will be obtained from all subjects and/or their legal guardian(s) that are interested in participating in the study. This will be done by the study coordinator and/or students who are GCP certified and have received the appropriate training on how to take informed consent.

Participants will be asked to attend a virtual or in-person consent visit, depending on regional COVID-19 restrictions.

### Additional consent provisions for collection and use of participant data and biological specimens

Clinical chemistry outcomes at baseline, week 1–2, 8, 10 and 16, will be captured from the participant’s nephrology clinic records or a requisition will be sent to participants directly from clinic. All samples will be collected and analyzed by Shared Health Diagnostics at both Health Sciences Centre and Seven Oaks Hospital.

### Confidentiality

The trial staff will ensure that the participants’ anonymity is maintained. The participants will be identified only by a participant ID number on all trial documents (except the email addresses on REDCap, remuneration forms, consent form and the study master list) and any electronic database. All documents will be stored securely and only accessibly by trial staff and authorized personnel. The trial will comply with The Personal Health Information Act (PHIA) or The Freedom of Information and Protection of Privacy Act (FIPPA) of Manitoba.

### Conflict of interest

The authors have no competing interests to declare.

### Access to data

In addition to the research team and individuals from their institutions (Chronic Disease Innovation Centre, Centre for Healthcare Innovation, Health Sciences Centre and University of Manitoba) direct access will be granted to authorized representatives from the Sponsor, host institutions and the regulatory authorities to permit trial-related monitoring, audits and inspections.

### Post-trial care

Participants will continue to be followed by their nephrologist post trial.

### Dissemination policy

A meeting will be held after the end of the trial to allow discussion of the main results among the collaborators prior to publication. The results of the trial will be submitted for to conferences for presentation and for publication in a peer reviewed journal.

## Discussion

The findings of this study may provide additional data to help inform dK recommendations in patients with CKD. This study will also help increase our understanding of how dietary restrictions are implemented and the effects these restritions can have on people’s quality of life. The findings of this study may also help inform other dietary interventions in CKD, such as the use of fruit and vegetables as dietary bicarbonate sources in the treatment of metabolic acidosis, for which the perceived risk of hyperkalemia may be a barrier for practioners and people living with CKD.

### Electronic supplementary material

Below is the link to the electronic supplementary material.

Supplementary Material 1

## Data Availability

Not applicable; this is a study protocol.

## Abbreviations

AE: Adverse event
ASA24: Automated Self-Administered 24-hour Canada dietary assessment tool
BMI: Body mass index
CKD: Chronic Kidney Disease
CRF: Case report form
dK: Dietary potassium
DSMB: Data Safety Monitoring Board
eGFR: Estimated glomerular filtration rate
ESRD: End stage renal disease
FIPPA: Freedom of Information and Protection of Privacy Act
GCP: Good clinical practices
K: potassium
KDQOL-SF: Kidney Disease Quality of Life Short Form
PHIA: Personal Health Information Act
PI: Prinicpal investigator
RAASi: Renin-angiotensin-aldosterone system inhibitors
RCT: Randomized controlled trial
RD: Registered dietitian
SGLT2i: Sodium-glucose cotransporter 2 inhibitors
sK: Serum potassium
STS5: Sit-to-stand five times
