# Supplementary material for: Dietary potassium liberalization with fruit and vegetables versus potassium restriction in people with chronic kidney disease (DK-Lib CKD): a clinical trial protocol
Source: BMC Nephrol. 2023 Oct 13;24:301. doi: 10.1186/s12882-023-03354-4 (PMC10576319; doi:10.1186/s12882-023-03354-4)

**Appendices**

**Appendix A: DK-Lib CKD Trial Flow Chart Appendix B: Automated Self-Administered 24-hour Canada (ASA24^®^) dietary assessment tool**

## Consent

**Screening/Baseline Visit**

**Excluded:**

- Individuals do not meet the inclusion /exclusion criteria
- Declined to participate

**Randomization sequence and Allocation (n: 30)**

20 participants at SOGH and 10 participants at HSC

CY

**Liberalized Dietary K Diet**

(n=15)

**Standard Dietary K Diet**

(n=15)

- Standard ongoing multidisciplinary CKD care
- Oral sodium bicarbonate therapy

## Data Analysis

**Liberalized Dietary K Diet**

(n=15)

6-weeks

**Safety assessment at week 1-2 (day 10 ± 3 days):**

- Clinical chemistry
- Blood pressure

**Outcomes assessed at baseline, 1-2, 8, 10 and 16 weeks**

- Chair stand time (STS5)
- Health related quality of life (KDQOL-SF)
- Clinical chemistry
- Blood pressure
- Dietary intake (ASA24®)

**Standard Dietary K Diet**

(n=15)

**2-week washout period and treatment switch**

CY

6-weeks

**2-week run-in on Liberalized dietary K diet**

| Three day 24-hour food Log  **Participant ID:** | | | | | |
| --- | --- | --- | --- | --- | --- |
| **Day 1:** | | | | | |
| Time | Location (home, restaurant, work, etc.) | Were you watching TV or on your phone? | Were you eating alone or with someone else? Please state who (partner, co-worker, etc.) | Food (please include all information such as method of cooking, ingredients, etc.) | Amount (in cups, tbsp., tsp., pcs, etc.) |
|  |  |  |  |  |  |
|  |  |  |  |  |  |
|  |  |  |  |  |  |
|  |  |  |  |  |  |
|  |  |  |  |  |  |
|  |  |  |  |  |  |
| Time | Location (home, restaurant, work, etc.) | Were you watching TV or on your phone? | Were you eating alone or with someone else? Please state who (partner, co-worker, etc.) | Food (please include all information such as method of cooking, ingredients, etc.) | Amount (in cups, tbsp., tsp., pcs, etc.) |
|  |  |  |  |  |  |
|  |  |  |  |  |  |
|  |  |  |  |  |  |
|  |  |  |  |  |  |
|  |  |  |  |  |  |
|  |  |  |  |  |  |
|  |  |  |  |  |  |
| Time | Location (home, restaurant, work, etc.) | Were you watching TV or on your phone? | Were you eating alone or with someone else? Please state who (partner, co-worker, etc.) | Food (please include all information such as method of cooking, ingredients, etc.) | Amount (in cups, tbsp., tsp., pcs, etc.) |
|  |  |  |  |  |  |
|  |  |  |  |  |  |
|  |  |  |  |  |  |
|  |  |  |  |  |  |

**Appendix C: Health Related Quality of Life (QOL) using the physical function domain of the Kidney Disease Quality of Life Short Form (KDQOL-SF) questionnaire**

**Appendix D: Trial Flow Design**


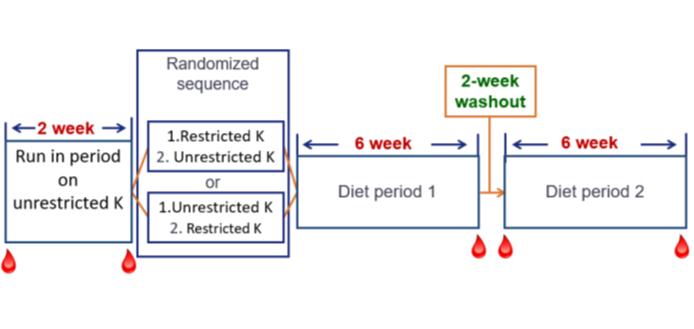

Supplement: Supplementary file 1 — Supplementary Material 1 [file 12882_2023_3354_MOESM1_ESM.docx]
